# Decision tools in health care: focus on the problem, not the solution

**DOI:** 10.1186/1472-6947-6-4

**Published:** 2006-01-20

**Authors:** Joseph Liu, Jeremy C Wyatt, Douglas G Altman

**Affiliations:** 1 Cancer Research UK/NHS Centre for Statistics in Medicine, Wolfson College, Oxford University, UK; 2 BHF Health Promotion Research Group, Department of Public Health, Oxford University, UK; 3 Health Informatics Centre, Dundee University, UK

## Abstract

**Background:**

Systematic reviews or randomised-controlled trials usually help to establish the effectiveness of drugs and other health technologies, but are rarely sufficient by themselves to ensure actual clinical use of the technology. The process from innovation to routine clinical use is complex. Numerous computerised decision support systems (DSS) have been developed, but many fail to be taken up into actual use. Some developers construct technologically advanced systems with little relevance to the real world. Others did not determine whether a clinical need exists. With NHS investing £5 billion in computer systems, also occurring in other countries, there is an urgent need to shift from a technology-driven approach to one that identifies and employs the most cost-effective method to manage knowledge, regardless of the technology. The generic term, 'decision tool' (DT), is therefore suggested to demonstrate that these aids, which seem different technically, are conceptually the same from a clinical viewpoint.

**Discussion:**

Many computerised DSSs failed for various reasons, for example, they were not based on best available knowledge; there was insufficient emphasis on their need for high quality clinical data; their development was technology-led; or evaluation methods were misapplied. We argue that DSSs and other computer-based, paper-based and even mechanical decision aids are members of a wider family of decision tools. A DT is an active knowledge resource that uses patient data to generate case specific advice, which supports decision making about individual patients by health professionals, the patients themselves or others concerned about them. The identification of DTs as a consistent and important category of health technology should encourage the sharing of lessons between DT developers and users and reduce the frequency of decision tool projects focusing only on technology. The focus of evaluation should become more clinical, with the impact of computer-based DTs being evaluated against other computer, paper- or mechanical tools, to identify the most cost effective tool for each clinical problem.

**Summary:**

We suggested the generic term 'decision tool' to demonstrate that decision-making aids, such as computerised DSSs, paper algorithms, and reminders are conceptually the same, so the methods to evaluate them should be the same.

## Backgound

### Decision support systems as a potential tool to enhance the uptake of evidence

Many problems facing health care systems today are not caused by lack of knowledge but by the gap between what we know and what we do in the face of staff shortages, economic pressures and rising public demand [1]. Systematic reviews or randomised-controlled trials of new health technologies published in prestigious journals are a linchpin of evidence based medicine and help to establish the effectiveness of drugs or procedures, but are rarely enough to ensure that the technology is actually used.

The process from innovation to routine clinical use is complex. For example, in cardiovascular disease prevention, despite the systematic reviews, evidence-based guidelines and decision tools (e.g. the Joint British Charts), there is continuing evidence to suggest that these approaches have not yet changed *actual *clinical practice[2,3]. The Leeds Acute Abdominal Pain system [4], which estimates patient-specific diagnostic probabilities and underwent extensive development and testing over decades, is scarcely used today. Many factors appear to influence the uptake of these systems, and the guidelines on which they are based[5]. For example, some health professionals are unaware of, or simply forget, guideline recommendations, while others fail to follow them because of patient choice or peer pressure.

Hundreds of computerised decision support systems (DSS) and other aids have been developed to assist patient management. In Garg et al's systematic review of controlled trials of DSSs, about two thirds of these are effective at narrowing knowledge gaps, improving decisions, clinical practice or patient outcomes [6], but many are not (e.g. computer-based guidelines on the management of angina and asthma[7]) Why did one third of the computerised DSSs that were sufficiently mature to be exposed to a randomised trial fail to influence clinical actions in Garg et al's systematic review [6]? Reasons why this might have happened include:

1. Failure of clinicians to use the DSS e.g. because they did not understand what it was for, the prevailing clinical culture was against it, their patients or peer group objected to it, it was too slow, or was not linked to the electronic patient record (EPR).

2. The DSS did not produce an effective output in time to influence their decision: e.g. the output was not available in time; they could not understand the output.

3. The output was not convincing enough to persuade the users to change their practice: e.g. the output showed poor accuracy, was badly worded, users had never before heard of this drug and required more details.

4. The output was available and was convincing enough to influence user decisions, but the user was unable to change their practice: e.g. the drug was too expensive to prescribe, there was adverse peer or patient pressure, the user was missing some vital information, equipment or skill that they needed before being able to enact their decision.

5. The performance of the clinicians was already optimal, given the circumstances and patient case mix.

Each of these potential reasons for failure needs to be considered carefully by DSS developers before they start work. This means that DSS developers need to start with the steps necessary to bring about the intended user actions or behaviour, not with the improvement of the quality of user decisions or the accuracy of the DSS itself. Those wishing to improve clinical practice and patient outcomes need to analyse the steps necessary to bring about the intended change and accept that, quite often, a DSS will not be the solution, as the long list of issues above demonstrates. We are thus advocating that the development of decision support systems need to shift from being technology led to problem led, and that a new mindset is needed to encourage this.

## Discussion

### Problems with current decision support systems

Some developers seem to construct technologically advanced systems with little relevance to the real world, while others create DSSs without first determining whether a clinical need exists[8,9]. We believe that there should be a move away from this technology-driven approach to one that entails identifying and employing the most effective method to improve practice, regardless of whether education, a high-tech personal digital assistant or a low-tech paper reminder is used.

Computerised DSSs (also called 'decision aids' [10]) are fundamentally no different from paper algorithms, nomograms, reminders or other aids to clinical decision-making, because they all aim to improve the appropriateness of clinical decisions, actions and patient health outcomes. Despite the concerns expressed above, we believe that this is an important class of health technology for which a consistent nomenclature is needed. We therefore suggest a generic term, 'decision tool', to demonstrate that these decision-making aids, which may seem very different from a technical perspective, are conceptually the same from a health technology assessment viewpoint. Examples of decision tools that do improve clinical practice include reminders for doctors,[11,12] patient information/support leaflets (e.g. O'Connor et al. [13]) and predictive scores (e.g. the paediatric logistic organ dysfunction (PELOD) score[14], the Ottawa ankle rules[15] and the Glasgow coma scale [16]). Computer-based reminder systems have been shown to be effective in increasing the use of preventive care in both inpatient and outpatient settings [6,12]. Some empirical evidence suggests that DSSs can have more impact than paper-based guidelines and checklists[17].

This article discusses problems with contemporary decision support systems and the need to adopt a decision tool 'mindset'. A formal definition of the term 'decision tool' and of their characteristics is given below.

Although good evidence exists for the clinical benefit of some DSSs, there are also numerous examples of failures and difficulties, for various reasons:

First, until recently, DSSs were rarely based on the best available knowledge. They should incorporate rigorous evidence, e.g. knowledge derived from well-designed, relevant studies or a large patient database.

Second, there has been insufficient emphasis on the need for the health professional or patient to capture high quality clinical data for the DSS.

Third, the development of DSSs has too often been technology-led. Their true role, of improving decisions and actions about individual patients, has frequently been ignored. A closely related issue is that the most appropriate method should be selected to overcome demonstrated barriers to change[5], avoiding what Gremy has termed the "idolatry of technology" by those working in medical informatics[18]. Some barriers require education or organisational change to abolish them, not a DSS at al[17].

Fourth, health technology assessment methods (such as studies on accuracy or impact, systematic reviews and economic analyses) have frequently been misapplied [18,19]. Correct application of these methods is necessary to evaluate their impact on clinical practice and their cost-effectiveness[20]. The cost-effectiveness of computer DSSs compared with paper-based decision tools has seldom been studied, and was missing from a recent large study on computerized reminders in US hospitals [12].

A fifth problem has been failure to address broader legal and ethical issues [21]. For example, health professionals using DSSs should always apply their own clinical judgement in the context of the patient and the encounter, and not unthinkingly follow its advice. The system should be designed to treat its user as a "learned intermediary" and not act as a black box[21,22].

A sixth problem is that developers and users of DSSs have too often failed to appreciate that effectiveness and cost-effectiveness will vary according to the user and their context.

Finally, DSS developers will need to become more aware of regulatory issues. Although DSSs are currently exempt from regulation, unlike the closed loop systems that measure patient variables and automatically adjust a drug infusion device for example, this may change[23]. For example, the National Institute for Health and Clinical Excellence in England is currently piloting methods to test the clinical and cost effectiveness of DSS[24]. If this pilot becomes a permanent NICE work programme, it will act as a regulatory hurdle to the introduction of DSS into the UK National Health Service.

Some of the above failures follow from insufficient clinical and patient involvement, due partly to our failure to recognise the role of different kinds of DSSs and their underlying similarities. However, this position is likely to change as more DSSs are used and some cause, rather than prevent, medical errors.

### Definition and characteristics of a decision tool

We propose the following definition of a decision tool: *A ****'decision tool' ****is an active knowledge resource that uses patient data to generate case-specific advice which support decision making about individual patients by health professionals, the patients themselves or others concerned about them*.

**Figure 1:**
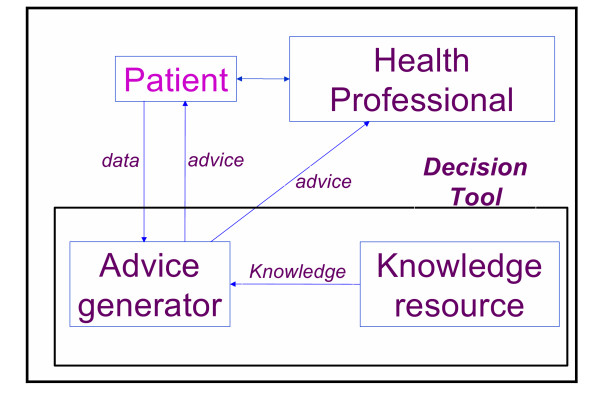
The role of a decision tool in shared decisions. One possible typology of decision tools.

This definition is an updated and more general version of Wyatt and Spiegelhalter's 1991 definition of computer decision aids ("active knowledge systems which use two or more items of patient data to generate case-specific advice")[20]. Figure 1 illustrates the generic role of a decision tool in the clinical consultation process and the flow of information between the patient, doctor and decision tool. The arrows in the figure represent pathways that information can take to and from the tool, doctor, patient, and diagnostic equipment.

In our model, decision tools have four important characteristics that can be readily transformed into criteria (Table 1):

**Table 1:** Assessment of a variety of aids to clinical decisions by decision tool criteria

	Characteristics of decision tools			
Tool	User Designed to aid clinical decisions by health professional or patient?	Target decision Decisions about a real individual patient?	Knowledge component Does tool use knowledge to assist interpretation or aid clinical decision making	Timing Is tool used before health professional or patient makes the relevant decision?

1. Computerized reminder system for preventive care (e.g. Dexter et al [12])	Yes	Yes	Yes	Yes
2. **Paper reminder to check for sign X, take into account symptom Y or take action Z when seeing a patient (e.g. Bryce et al [26] and Eccles et al [11])**	**Yes**	**Yes**	**Yes**	**Yes**
3. Care pathway (e.g. Holtzman et al [27])	Yes	Yes	Yes	Yes
4. **Tool to enhance shared decision making [28]**	**Yes**	**Yes**	**Yes**	**Yes**
5. Computerised patient interviewing (checklist for patient to complete, after which the data are presented to the doctor in summary form (e.g. Lilford et al [29])	Yes	Yes	Yes	Yes
6. **Sheet for doctor giving definitions of clinical findings in acute abdominal pain or advice on how to elicit them**	**Yes**	**Yes**	**Yes**	**Yes**
7. Nomograms	Yes	Yes	Yes	Yes
8. **Joint British Societies coronary risk prediction charts [2]**	**Yes**	**Yes**	**Yes**	**Yes**
9. Telemedicine system [30]	Yes	Yes	Yes	Yes
10. **Information leaflet for patient with acute abdominal pain**	**Yes**	**Yes**	**Yes**	**Yes**
11. Sheet summarising results of special investigations with advice on interpreting results	Yes	Yes	Yes	Yes
12. **Distance learning material used away from patients (e.g. in a course, or self-study) (see examples in Davis et al [31])**	**Yes**	**No**	**Yes**	**No**
13. Monthly performance feedback report (i.e. giving doctors feedback about their performance on previous groups of patients)	Yes	No	No	Yes
14. **Computer simulator to help doctors develop their diagnostic skills (e.g. Hoffer & Barnett [32])**	**Yes**	**No**	**Yes**	**Yes**
15. Imaging investigation, e.g. ultrasonography, computed tomography	Yes	Yes	No	Yes
16. **Laboratory test, e.g. white cell count, C-reactive protein**	**Yes**	**Yes**	**No**	**Yes**
17. Audit on clinical activities in a GP surgery	No	No	No	No

#### i) The target decision maker: the tool is designed to aid a clinical decision by a health professional and/or patient

This characteristic highlights the importance of shared decision-making between health professionals and patients. Decision aids for health professionals and patients are both included. If the patient is unable to make an informed decision (e.g. a child or someone in a coma), then a carer or relative familiar with his or her condition is an appropriate proxy.

#### ii) The target decision: the decisions concern an individual patient

The focus is on decisions about an identified individual patient, rather than on groups of patients (e.g. to support health policy) or on hypothetical patients (e.g. for teaching purposes).

#### iii) The knowledge component: the tool uses patient data and knowledge to generate an interpretation that aids clinical decision-making

A decision tool must contain some embedded knowledge to help a health professional or patient use patient data to generate an interpretation or aid to decision-making. Examples include: explicit advice, such as a printed recommendation for a course of action; interpretation, such as an asterisk meaning "this result is abnormal" or a predicted probability of death for an ICU patient; and reminders or alerts, such as "This patient is allergic to penicillin."

#### iv) Timing: the tool is used before the health professional or patient takes the relevant decision

A tool used retrospectively, after the relevant decision has been finally taken, is excluded. However, tools that interpret patient data such as a test result after a clinical encounter can still be classified as decision tools if their output is used to inform a later decision, for example to help the patient manage their own disease at home or during the next clinical encounter.

### Possible objections to the label "Decision tool"

Our proposed term, decision tool, includes the classical computer based decision support systems. Those developing DSSs might reject our blanket category, claiming significant differences between subclasses of these systems (e.g. how a specific tool works or is developed), in the same way that a chemist will recognise molecular differences between the individual drugs that form a single therapeutic class. However, from certain clinical and health policy perspectives, such differences are often irrelevant, as is often the case with drugs from the same therapeutic class.

We also disagree with some technologists[25] and believe there is essentially no difference between the methods used to evaluate a new drug or a new decision tool. While qualitative methods are necessary to help elucidate barriers to change, requirements for a decision tool or reasons for failure, there is no alternative to rigorous study designs such as the randomised controlled trial to reliably quantify a tool's impact on clinical decisions, actions or patient outcomes. Systematic reviews demonstrate the feasibility of conducting RCTs, for example Garg et al found one hundred RCTs of computer based DSS [6].

### Examples of decision tools

Table 1 provides examples of tools with and without the above characteristics. A care pathway (Example 3) is a pre-printed record designed to aid health professionals in recording data and interpreting them as well as in making decisions (fulfilling characteristics 1 and 4) about an individual patient (characteristic 2). It is a knowledge resource for health professionals that enables them to actively use patient data to make decisions (characteristic 3). Clearly, care pathways are decision tools.

**Figure 2:**
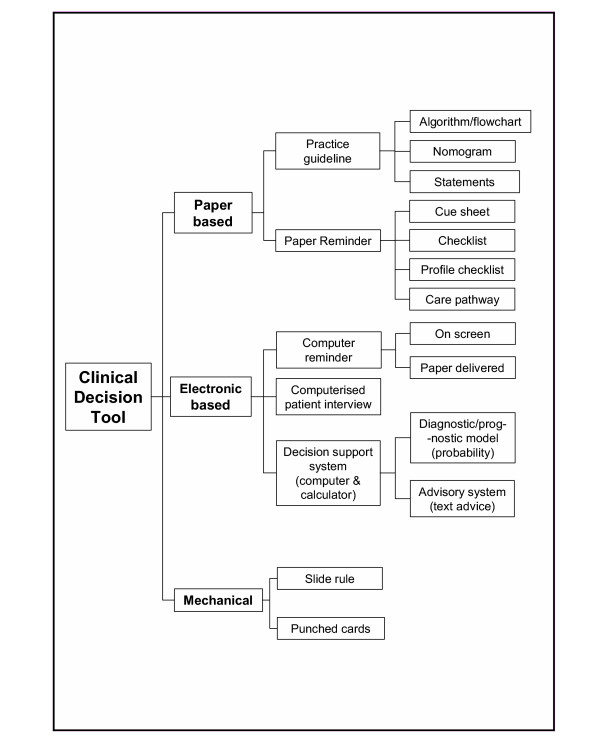
One possible typology of decision tools.

Some examples of aids that are not decision tools include distance learning material used away from patients (Example 12) and imaging investigations/laboratory tests (Examples 15 and 16), which are not knowledge resources (characteristic 3). However, an algorithm or other tool to support interpretation of the results of such tests is a decision tool. For example, a sheet summarising test results would be included if it included knowledge on how to interpret the results and obtain predictions that inform patient management. Some examples depend on the user and current task. For example, a computer-based simulator used to help physicians develop their diagnostic skills would not be a decision tool if the data they enter are not about a patient they are managing (Example 14). However, it is a decision tool if they enter data about a real patient. An audit of a GP surgery's clinical activities fails to have any of the characteristics and is not a decision tool (Example 17). The typology in Figure 2 shows one way to visualise the diversity of decision tools.

### Recommendations

We have argued that DSSs and other computer-based, paper-based and even mechanical clinical decision aids are members of a wider family that we call decision tools. By viewing decision tools as a group their role in health care becomes clearer, which should encourage clinical involvement in developing such tools and evaluating their impact on clinical practice. The excessive emphasis on technology to date has probably resulted from lack of balance in the involvement of clinicians and informatics experts or computer scientists, some of whom did not appreciate the crucial need for input from clinicians and epidemiologists in the development and testing of these tools. Equally, it is likely that most clinicians do not appreciate the potential of these tools as a crucial step in helping implement the evidence form rigorous studies. The identification of decision tools as a coherent and important category of health technology should encourage the involvement of clinicians and sharing of lessons between decision tool developers and users, reduce the frequency of decision tool projects focusing only on technologies, and reduce silo thinking by those in both clinical and informatics disciplines.

We believe that the focus of evaluation should thus become more clinical. For tools that are designed to help improve clinical practice – as opposed to exploring some technical issue – it is not sufficient to evaluate the accuracy of the tool compared with routine clinical practice or a gold standard[20]. Rather, its impact should be evaluated against other computer, paper- or even mechanical tools, in order to identify the most cost-effective tool for each clinical problem. It is unlikely that the most cost-effective option will always be computer-based.

How should adoption of this decision tool mindset be encouraged? Authors and editors could be encouraged to use the term in titles and abstracts. We propose the inclusion of 'decision tool' as a new Medical Subject Heading (MeSH) term to aid the identification of empirical studies for clinical and research purposes. A joint clinician and decision tool developers' network could be established, with an infrastructure including e-mail lists, web support materials, conferences and a co-ordinating resource centre. Finally, a case can be made for multidisciplinary R&D programmes on decision tools, jointly supported by clinical and informatics funding bodies. Table 2 illustrates our viewpoint by contrasting the "old think" of many DSS projects with the approach that makes sense for those who wish to consider using decision tools as a promising technology to help get evidence into practice.

**Table 2:** The contrast between the "old think" of many DSS projects and the "new think" that considers decision tools as a potential technology to help get evidence into practice.

**Old think**	**New think**
Complex computerised decision support system that demands respect and understanding	A discrete decision tool with the complexity concealed within a simple functional exterior
Hard technical problems are the main focus	Hard knowledge management problems are the main focus
Evaluation should focus on the DSS	Evaluation should focus on the knowledge problems
The technology dominates the project	The problem dominates the project
Select problems that DSS can address	Focus on a knowledge problem and match the solution to this – ignoring DSS if need be
Improved decisions are the end point	Getting evidence into practice is the end point
The important distinctions are between DSS reasoning methods	The important distinctions are between different knowledge management problems and their causes

## Summary

Some developers of DSSs have constructed technologically advanced systems with little relevance to the real world, while others created DSSs without first determining whether a clinical need exists. Computerised DSSs are fundamentally no different from paper algorithms, nomograms, reminders or other aids to clinical decision-making, because they all aim to improve the appropriateness of clinical actions and patient health outcomes. We therefore suggest the generic term, 'decision tool', to demonstrate that these decision-making aids, which may seem very different from a technical perspective, are conceptually the same from a clinical viewpoint. A decision tool is an active knowledge resource that uses patient data to generate case specific advice, which support decision making about individual patients by health professionals, the patients themselves or others concerned about them. The impact of computer-based decision tools should be evaluated against other computer, paper- or even mechanical tools, in order to identify the most cost effective option, which is unlikely to be always computer-based.

## List of abbreviations

DSS: Decision support system

## Competing interests

The author(s) declare that they have no competing interests.

## Authors' contributions

JL had the idea for the article and wrote the manuscript with contributions from JCW and DGA. All authors read and approved the final manuscript.

## Pre-publication history

The pre-publication history for this paper can be accessed here:


